# Circulating microRNA profiles of Hendra virus infection in horses

**DOI:** 10.1038/s41598-017-06939-w

**Published:** 2017-08-07

**Authors:** Christopher Cowled, Chwan-Hong Foo, Celine Deffrasnes, Christina L. Rootes, David T. Williams, Deborah Middleton, Lin-Fa Wang, Andrew G. D. Bean, Cameron R. Stewart

**Affiliations:** 1 CSIRO Australian Animal Health Laboratory, Geelong, Victoria Australia; 2 Programme in Emerging Infectious Diseases, Duke-NUS Medical School, Singapore, Singapore

## Abstract

Hendra virus (HeV) is an emerging zoonotic pathogen harbored by Australian mainland flying foxes. HeV infection can cause lethal disease in humans and horses, and to date all cases of human HeV disease have resulted from contact with infected horses. Currently, diagnosis of acute HeV infections in horses relies on the productive phase of infection when virus shedding may occur. An assay that identifies infected horses during the preclinical phase of infection would reduce the risk of zoonotic viral transmission during management of HeV outbreaks. Having previously shown that the host microRNA (miR)-146a is upregulated in the blood of HeV-infected horses days prior to the detection of viremia, we have profiled miRNAs at the transcriptome-wide level to comprehensively assess differences between infected and uninfected horses. Next-generation sequencing and the miRDeep2 algorithm identified 742 mature miRNA transcripts corresponding to 593 miRNAs in whole blood of six horses (three HeV-infected, three uninfected). Thirty seven miRNAs were differentially expressed in infected horses, two of which were validated by qRT-PCR. This study describes a methodology for the transcriptome-wide profiling of miRNAs in whole blood and supports the notion that measuring host miRNA expression levels may aid infectious disease diagnosis in the future.

## introduction

Hendra virus (HeV) is a negative strand RNA virus belonging to the genus *Henipavirus* family *Paramyxoviridae*. Its geographical range spans most of the north-eastern coast of Australia, ultimately determined by the range of its natural reservoir hosts, pteropid fruit bats (flying foxes)^1^. HeV was discovered in 1994 during an outbreak of severe respiratory disease at a horse stable in Hendra, Queensland^2^. HeV was confirmed as the etiological agent of this outbreak that resulted in the death of 14 horses and one person. Between 1994 and 2010 HeV outbreaks occurred sporadically in coastal Queensland and north-eastern New South Wales. Between 2011 and 2013, however, 34 HeV incidents were reported in horses, increasing public concern over HeV as an emerging infectious disease. A HeV vaccine was developed for use in horses in 2012^3^, and since 2013 reported incidents have declined but continue at a reduced level and in unvaccinated horses. Seven human cases of HeV disease have also occurred, four of which resulted in fatal disease.

To date, all cases of HeV disease in humans have resulted from close contact with infected horses, with virus transmission resulting from contact with an infected horse’s nasal discharge, blood, saliva or urine. Infected horses do not display signs of disease (for example, elevated heart rate, temperature) until after viral genome is detectable in secretions, and interaction between owners or veterinarians and infected horses increases with the onset of florid diseases and viral peak shedding^4^. Accordingly, horses pose a transmission risk both late in the incubation period and also after they become clinically ill. An assay that could identify infected horses early in the infection course, prior to the onset of high levels of virus shedding, would reduce the risk of zoonotic HeV transmission by assisting decision-making around in-contact animals during management of an HeV outbreak. Since the clinical signs of HeV disease in horses are not specific and the associated pathological changes are not pathognomonic, laboratory diagnosis is essential. Current laboratory testing to confirm suspected cases of HeV disease in horses includes virus isolation, detection of viral RNA in clinical or post-mortem specimens or viral antigen detection in tissues taken at necropsy^5^. Serological tests, such as ELISA and virus neutralization test, are also available but are of limited use for the diagnosis of acutely infected horses prior to the development of antibodies.

MicroRNAs (miRNAs) are a class of single-stranded non-coding RNAs that regulate biological processes in eukaryotes^6–10^. The production of miRNAs requires several processing steps, first where primary miRNAs (pri-miRNAs) are cleaved by the ribonuclease Drosha to produce precursor miRNAs (pre-miRNAs) which in turn are cleaved by the ribonuclease Dicer to produce mature, single stranded miRNAs^11, 12^. The binding of miRNAs to target gene mRNA can lead to the degradation, suppression or even the up-regulation of target mRNA levels. The regulation of biological processes by miRNAs often involves changes in miRNA transcript levels. Thus miRNA expression profiles are emerging as valuable tools to assist disease diagnosis, including for viral infections^13, 14^. For diagnostic decision support, miRNAs are attractive for several reasons – they can be detected from easily-collected bodily fluids such as blood and urine, are stable once secreted into bodily fluids, and can be detected using common and highly sensitive molecular diagnostic techniques such as PCR^15^.

We have previously shown that infection of human cells with HeV changes the expression levels of several host-encoded miRNAs, including miR-146a, which was also shown to be up-regulated in the blood of three horses experimentally infected with HeV^16^. The up-regulation of miR-146a in horse blood precedes the detection of viral genome in the same samples by several days, supporting the notion that miRNA profiling could be used to aid early HeV disease diagnosis. However, as miR-146a is induced by several pathogens, including bacteria^17^ and multiple viruses^18, 19^, the development of miRNAs as disease-specific biomarkers likely requires a more comprehensive measurement of miRNAs. In this study, we describe a methodology for the transcriptome-wide identification, quantification and differential expression analysis of miRNAs in horse blood. Our study identifies a number of miRNAs differentially expressed in the blood of horses infected with HeV, and supports the further development of miRNA profiling to aid henipavirus disease diagnosis.

## Results

### MiRNA identification, quantification and normalization

**Figure 1:**
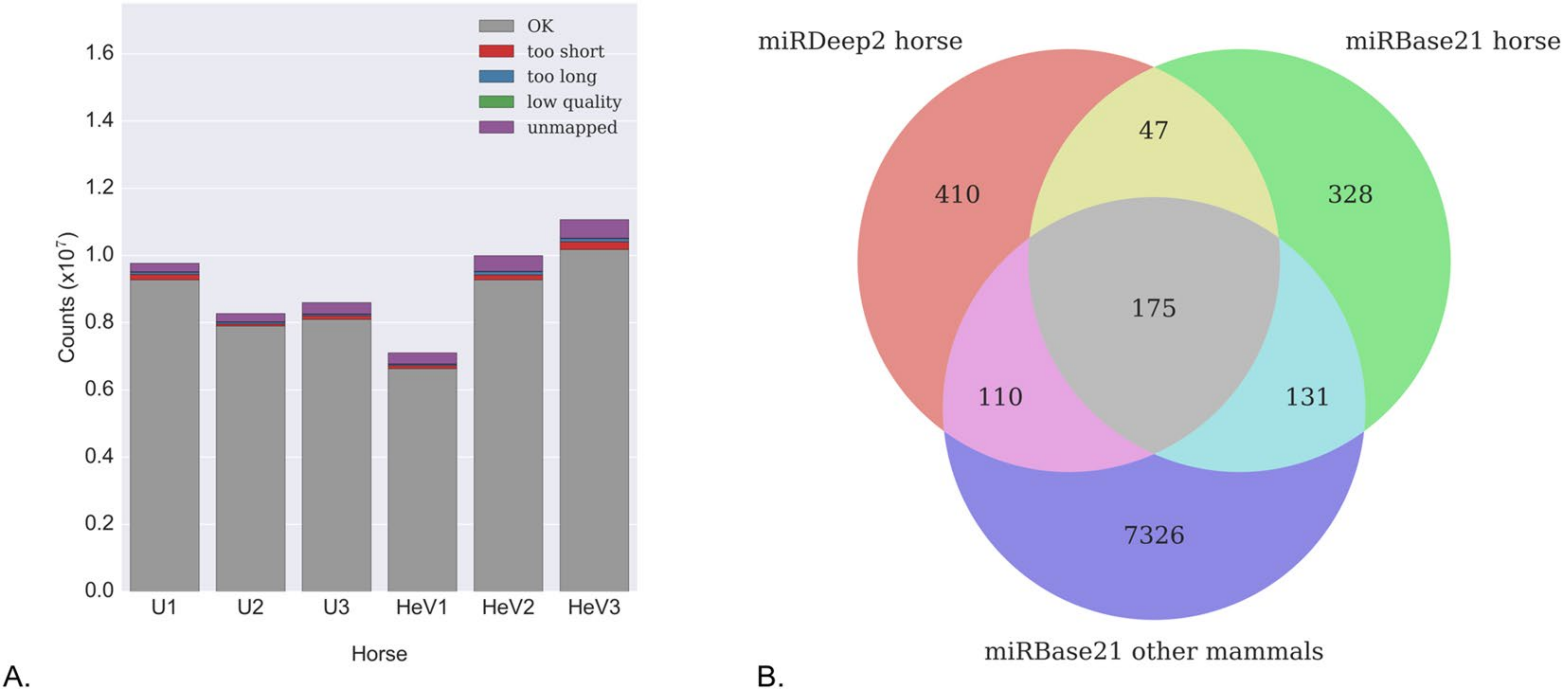
(**A**) Quality control and filtering of raw reads from samples analyzed in this study. U1, U2 and U3 refer to samples derived from uninfected horses, while HeV1, HeV2 and HeV3 refer to samples from HeV-infected horses listed in Table 1. Unique read counts per sample were: U1:26207, U2:25007, U3:38479, HeV1:32102, HeV2:50257, HeV3:64798. (**B**) Comparison between horse miRNAs sequenced in this study (miRDeep2 horse) and reference miRNAs from miRBase21 (miRBase21 horse, miRBase21 other mammals). The majority (410) of miRNAs detected in horse blood were unique from miRNAs listed in miRBase, whilst 332 candidates matched miRNAs cataloged in miRBase.

Whole blood samples from three horses infected with HeV were submitted to the CSIRO Australian Animal Health Laboratory for diagnostic testing (Table 1). HeV was isolated from whole blood of two of the three horses (horses 2 and 3), and viral genome was detected in all three. All three infected horses were seronegative and were therefore likely sampled during the early stages of infection. Three healthy seronegative control horses were sampled from a single property in Victoria, Australia, where HeV is not considered endemic. EDTA blood from the control horses were negative for viral genome by PCR. Small RNA deep sequencing resulted in 7–11 million raw reads obtained per sample, which have been submitted to the NCBI short read archive (SRA) BioProject PRJNA323953. Reads were trimmed of adaptors and filtered, resulting in a loss of 4.5–8% of raw reads, leaving 6–10 million reads per sample for further analysis (Fig. 1A). The majority (92–95%) of sequences were deemed high quality by FASTQC.

**Table 1:** Diagnostic test results for horses infected with HeV analyzed in this study.

Location	Virus isolated?^5^	HeV M gene TaqMan assay (PCR)^37^	Indirect ELISA for detection of antibodies to HeV^38^
NSW, Australia	No	Positive (C_t_ 33.0)	Negative
NSW, Australia	Yes	Positive (C_t_ 27.1)	Negative
QLD, Australia	Yes	Positive (C_t_ 29.2)	Negative

MiRDeep2 was used to identify all known and novel miRNA transcripts amongst the pooled data for the six samples (Supplementary Table 1). MiRNAs with sequences matching those deposited in miRBase were accepted and included for further analysis. Novel candidate miRNAs were also included if they had a miRDeep2 score ≥1, consistent with previous studies^20, 21^. A total of 742 different mature miRNA transcripts were detected, corresponding to 593 different precursors (5p and 3p miRNAs were counted separately) (Fig. 1B). A total of 332 miRNAs in horse blood matched known miRBase entries. Of those, 47 appear to be horse-specific (yellow region), while 110 are conserved between horses and other mammals but have not been previously identified in horse (pink region). The remaining 410 candidates (red region) are putative novel miRNAs. We did not observe a significant difference in the total number of miRNAs identified in infected versus uninfected horses (data not shown).

**Figure 2:**
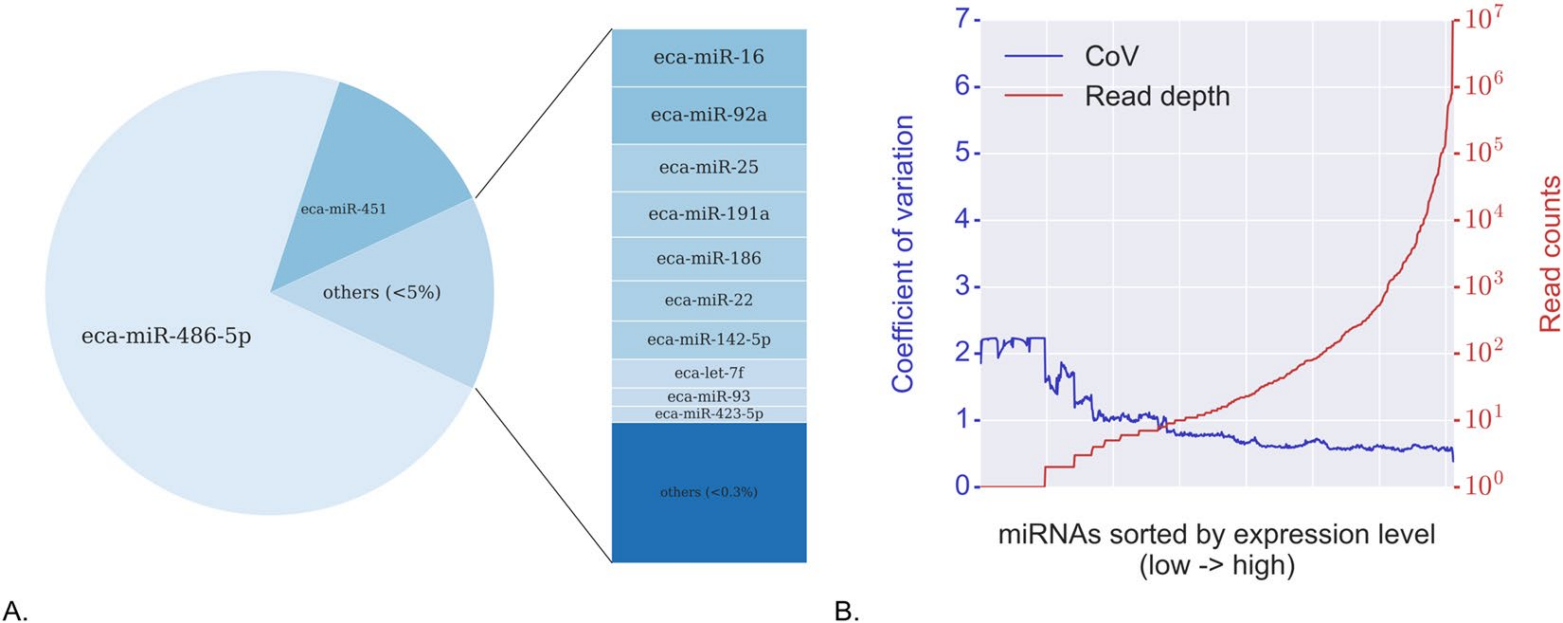
(**A**) The relative composition of miRNAs detected in infected and uninfected horse blood samples. Approximately 73% of all miRNA reads across the six samples corresponded to miR-486-5p, which is highly expressed in erythrocytes. miRNAS representing between 0.3–5% of total reads are drawn as a stacked bar to the right of the pie. The remaining miRNAs (“others <0.3%”) each took up less than 0.3% of the total read pool, corresponding to a cutoff point of about 125,000 actual reads. (**B**) Inter-sample variance (blue line) decreases with increasing depth of sequencing (red line). MiRNAs with <10 counts across the six samples had on average very high inter-sample variance.

Read counts were determined for each mature miRNA transcript (Supplementary Table 2). Total counts included all reads that mapped to a locus (as opposed to reads matching the canonical/consensus sequence only), and care was taken to avoid counting reads more than once. By examining the combined counts for the six samples, horse blood was shown to be heavily skewed towards a small set of dominant miRNAs (Fig. 2A). MiR-486–5p was extremely abundant compared to any other miRNA, taking up approximately 73% of all sequencing reads in the combined samples. Approximately 13% of the total reads corresponded to the second most abundant miRNA, miR-451, while miR-16 and miR-92a each took up about 1.5% of reads. Other highly expressed miRNAs in horse blood included miR-25, miR-191a, miR-186, miR-22 and miR-142-5p.

To examine the relationship between abundance and inter-sample variance, miRNAs were sorted by increasing read depth and plotted on a log_10_ scale against their coefficient of variation (CoV) (Fig. 2B). The CoV graph revealed a relatively clear relationship between read depth and variance. The point at which CoV dropped below 1.0 corresponded to a read depth of approximately 10^1^ (i.e. 10) reads, and therefore data for miRNAs with <10 reads should be considered qualitative only. A total of 41 miRNAs were observed exclusively in infected horses, of which five had reads in all three infected samples. A total of 24 of these were singletons (only one read detected), while four had ≥10 reads (maximum reads 21). A further 20 miRNAs were present only in uninfected horses, however only one displayed reads in all three uninfected horses. Nine of these 20 were singletons and none had ≥10 reads (maximum 9). Based on these small counts, it appeared that the presence/absence of specific miRNAs is unlikely to be a robust measure of HeV infection status. We therefore sought to identify quantitative differences in miRNA abundance between the infected and uninfected animals.

**Figure 3:**
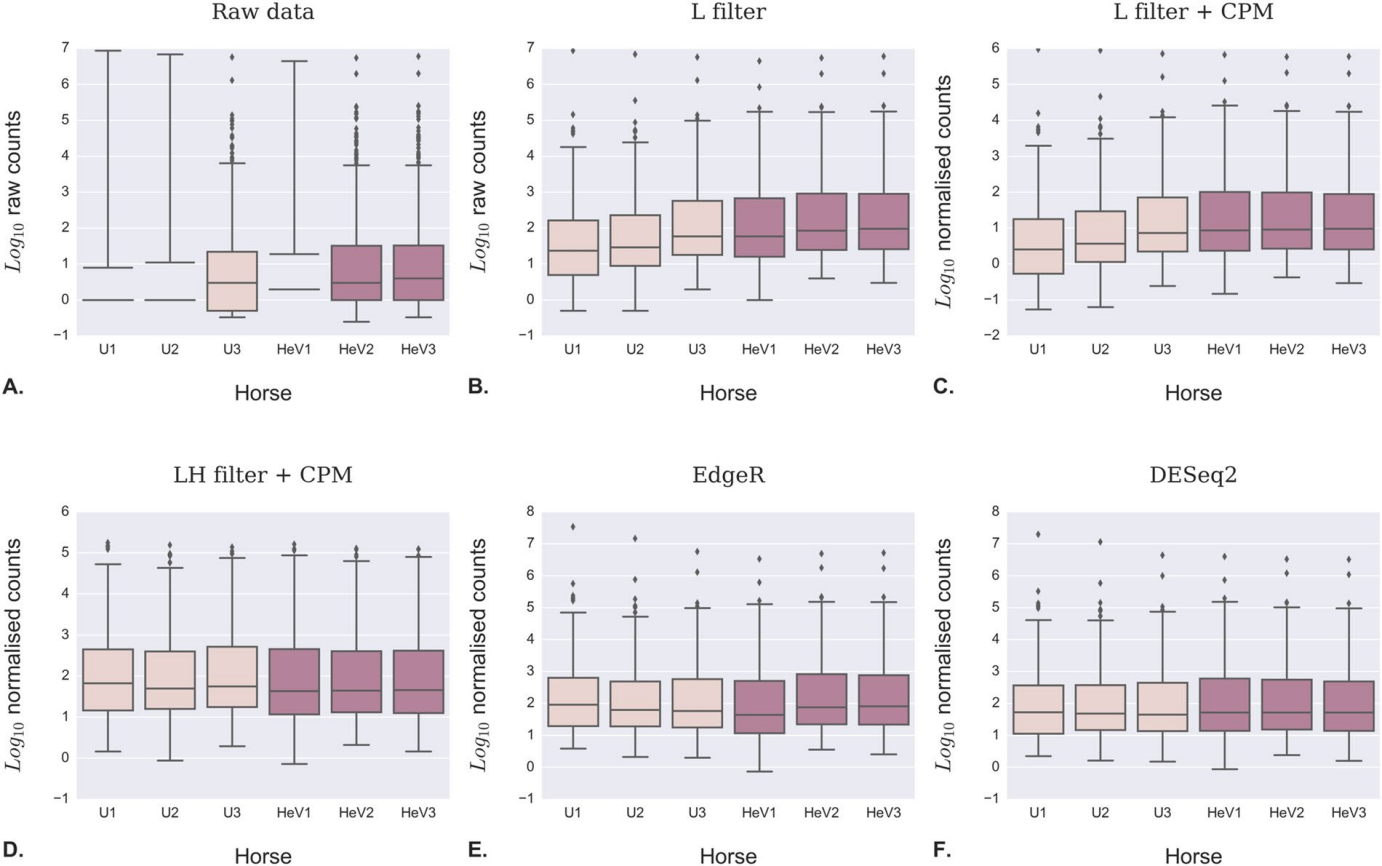
The impact of different data normalization strategies on miRNA profiles. Boxplots illustrate the impact of various normalization strategies on inter-sample variance. The midline of each box is the median value (50^th^ percentile), the upper and lower bounds of the box represent the interquartile range (25^th^ to 75^th^ percentiles), while the whiskers represent 1.5x the interquartile range. Values outside this range are considered outliers and are plotted individually as dots. Data are plotted on a log_10_ scale. Distributions are shown for raw reads (**A**, raw data), raw reads excluding low read counts (**B**, L filter), data as in (**B**) normalized for counts per million (**C**, L filter + CPM), data as in (**B**) excluding both low and high read counts (**D**, LH filter + CPM), (**E**) data as in (**B**) analyzed by EdgeR, (**F**) data as in (**B**) analyzed by DESeq2. *Note regarding Fig. 3A (raw data): A large number of miRNAs with low abundance are still present within the raw data including many that were not detected in all six samples. Consequently, the 25^th^ percentiles for U1, U2, and HeV1 were equal to zero, causing the boxplot drawing algorithm to fail. Removal of low-abundance miRNAs resolved the problem (3**B**–**F**).

Technical artifacts caused by library preparation and sequencing were corrected by normalization in order to perform quantitative cross-sample analysis (Fig. 3). Between-sample differences were evident in the raw counts (Fig. 3A, Raw data), and removing miRNAs with low abundance made a clear improvement in inter-sample variance (L filter). Global normalization (counts per million, CPM) made very little discernable improvement over L filter alone, whilst removal of both low count miRNAs and the two over-abundant outliers (miR-486 and miR-451) followed by CPM normalization led to a greatly improved profile (LH filter + CPM). Discarding low-count miRNAs followed by EdgeR or DESeq2 normalization produced horizontally aligned medians and consistent interquartile ranges, without having to discard the highly abundant miRNAs. We therefore elected to normalize data using the DESeq. 2 normalization method in subsequent analyses.

**Figure 4:**
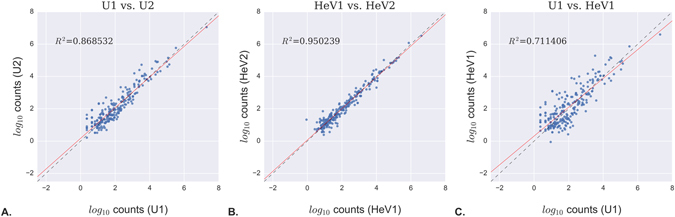
Representative inter-sample comparisons at the single-miRNA level. (**A**) Uninfected horse #1 vs. uninfected horse #2. (**B**) Infected horse #1 vs. infected horse #2. (**C**) Uninfected horse #1 vs. infected horse #1. Points represent DESeq2-normalized read counts for each miRNA and are drawn in log_10_ scale on both axes. The midline ($\mbox{-}  \mbox{-}  \mbox{-}$) illustrates optimal correlation (i.e. points that fall on this line have equal expression in both samples). In contrast, points that lie further from the midline represent between-sample variation for individual miRNAs. *R* ^*2*^ values reflect the overall correlation between samples, while the line of best fit (red line) was determined using linear least-squares regression.

To further evaluate inter-sample consistency following normalization, we performed pairwise analyses at the single-miRNA level. The blood miRNA profile of two uninfected horses showed a high degree of similarity, as did the profiles of two infected horses (Fig. 4A and B, respectively). By contrast, the profile of an uninfected horse showed a lower degree of similarity to an infected horse, as reflected by a lower correlation coefficient (Fig. 4C). Similar trends were observed for other horses (e.g. U1 vs. U3, HeV1 vs. HeV3, U2 vs HeV2, data not shown). These data suggest that HeV infection induces changes in the miRNA profile of horse blood that can be detected above the background level of biological and technical variation.

### Differential expression analysis

**Figure 5:**
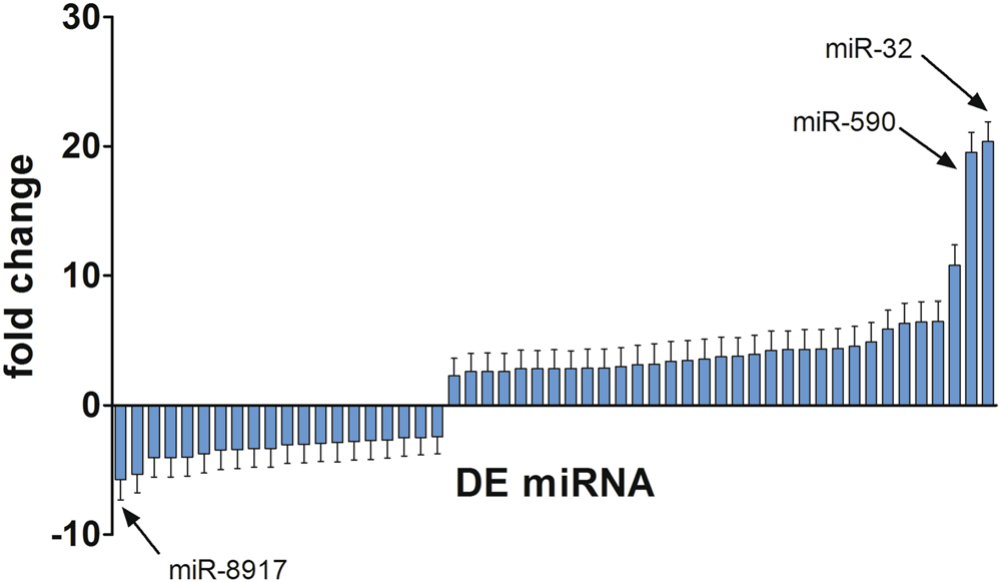
Normalized fold change for miRNAs upregulated or downregulated in infected horses as determined by DESeq2. Each bar represents a miRNA whose expression level was significantly different (FDR < 0.05) between the groups of infected and uninfected horses. Error bars represent standard error.

We next sought to identify miRNAs with significantly altered expression levels between infected and uninfected horses. By using DESeq2 to perform count-based differential expression (DE) testing based on the negative binomial distribution^22, 23^, a subset of miRNAs were identified that were either upregulated or downregulated in infected vs. uninfected animals (Fig. 5, Supplementary Table 3). EdgeR yielded a similar set of DE miRNAs (Supplementary Table 4). In total, 37 miRNAs were significantly DE according to both methods, of which 18 were up-regulated (elevated in infected horses) and 19 were down-regulated.

**Figure 6:**
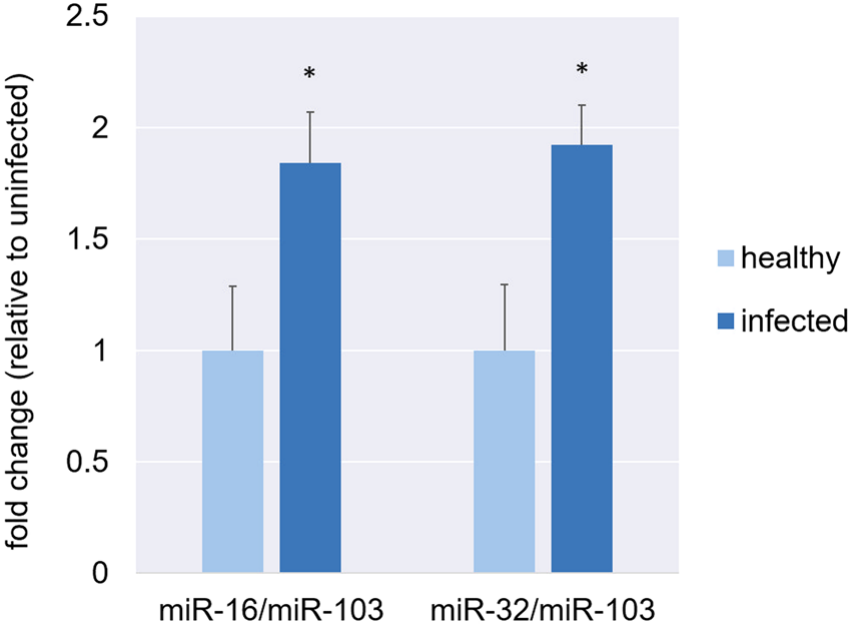
qRT-PCR validation of two miRNAs identified as differentially expressed by miRNA profiling. Graphs show relative expression levels of miR-16 and miR-32 in infected and uninfected samples. Data is normalized against relative levels of miR-103. Values were normalized to set the average for uninfected horses to 1. The difference between infected and uninfected groups was analysed in Graph Pad Prism software, using a two-tailed Student’s *t* test, with a *P* value of <0.05 considered to be statistically significant. Error bars represent standard deviation of triplicates.

A second approach was to identify DE miRNAs based on miRNA ratios, analogous to the use of housekeeping genes as internal normalization controls in quantitative real-time PCR. In contrast to evaluating the quantity of an individual miRNA in isolation, this strategy considers the ratio of two different miRNAs to determine whether there is a significant difference between the average ratio for the infected group and that of the uninfected group. MiR-103 was selected as an initial housekeeping miRNA based on previous studies^24^ and our own observation that miR-103 expression levels were not significantly different between infected and uninfected horses (data not shown). Two miRNAs significantly upregulated in infected horses, as identified by DESeq2 (miR-16 and miR-32), were also upregulated when expression levels were measured by qRT-PCR and normalized against relative levels of miR-103 (Fig. 6). This data validates two results from the miRNA profiling pipeline.

**Figure 7:**
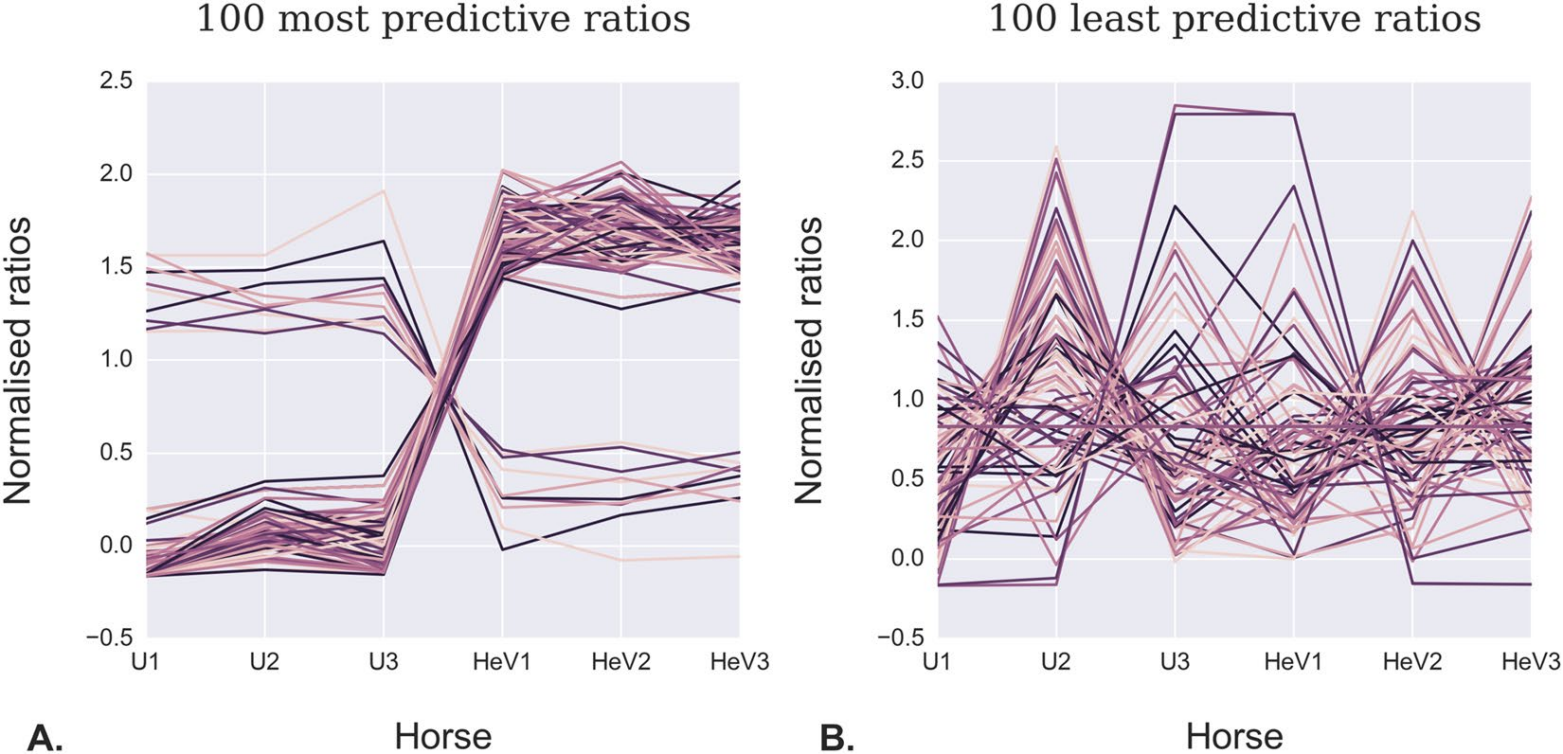
Ratios of pairs of miRNAs with the potential to distinguish infected from uninfected horses. All possible combinations of miRNA pairs were tested, and the (**A**) 100 most-predictive, and (**B**) 100 least-predictive miRNA pairs are illustrated.

We next assessed the miRNA-Seq data to identify ratios of particular miRNA pairs which together would enable the best possible distinction between infected and uninfected horses. To this end, every possible combination of miRNAs were tested in order to identify pairs whose ratios produce the most statically significant differences between the two groups (i.e. the highest signal to noise ratio). By taking the ratio of $\frac{miRNA\_A}{miRNA\_B}$ for each horse individually, inter-replicate differences were minimized. The results were ranked by corrected p-value and the top-ranking miRNA pairs identified (Fig. 7, Supplementary Table 5). After correcting for multiple testing using the false discovery rate (FDR) method, we note that the corrected p-values were improved over the raw p-values obtained for individual miRNAs in isolation. This strategy therefore appeared to improve the signal to noise ratio, and we noted that many of the miRNAs identified in this way had also been individually identified as differentially expressed miRNAs.

### IsomiR analysis

**Figure 8:**
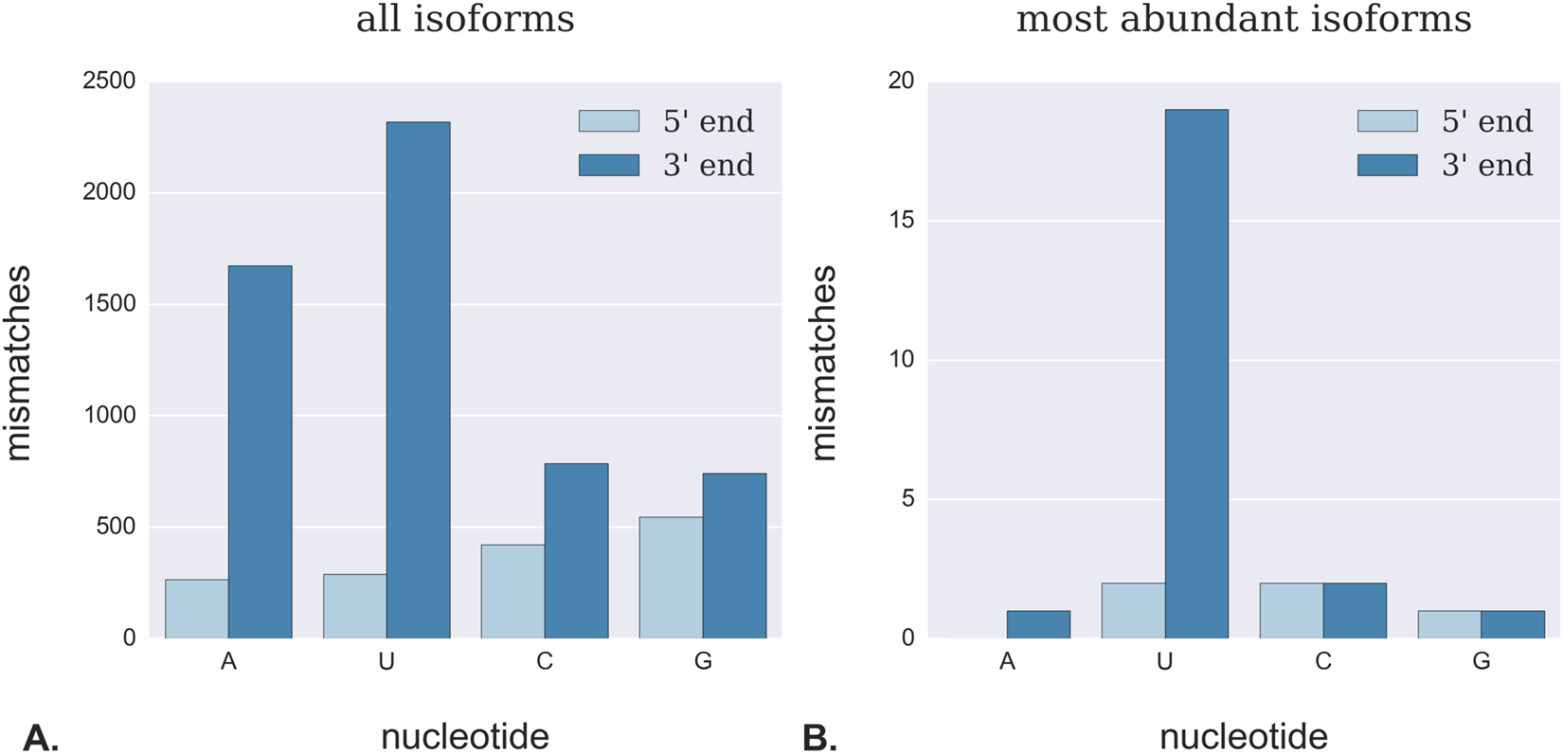
Approximately one in five reads featured a nucleotide mismatch relative to the reference horse genome. (**A**) Amongst the total collection of reads detected in the six samples (45174 different sequences), mismatches at the 3′ end of the read were considerably more abundant than mismatches at the 5′ end. Furthermore, mismatching 3′ nucleotides were more likely to be either uridine (U) or adenine (A) than cytosine (C) or guanine (G). (**B**) When the most-abundant isoforms of each miRNA (300 different sequences) were considered separately, a similar trend was observed but with a dramatic increase in the proportion of mismatches due to non-templated 3′ U additions.

Downstream application of transcriptome-wide miRNA profiling may include PCR-based detection for select miRNAs. As PCR-based detection can be highly sensitive to sequence variation, we next examined sequence variation amongst individual reads mapping to each miRNA locus. Furthermore, we assessed whether the most-abundant isomiR might be DE while pooled isoforms were not, or vice versa. We therefore sought to identify the most highly-abundant isoform corresponding to each locus (Supplementary Table 6). Most-abundant isoforms with >10 reads were identified for 271 mature and 29 star miRNAs. Of these, 65 (22%) had a nucleotide mismatch relative to the consensus sequence as reported by miRDeep2. We observed that 62 (21%) of the most-abundant isoforms had a nucleotide mismatch relative to the reference genome, and 19 of these (6.3% of all mRNAs, 20% of all mismatches) had a non-templated 3′ uridine (U) addition (Fig. 8). Of the remaining most-abundant forms containing a mismatch relative to the reference genome, 12 exhibited a mismatch within the seed region, including eca-miR-147b plus 11 of the novel miRNAs. Finally, we performed DESeq2 differential expression analysis using count data for only the most-abundant isoform of each miRNA (Supplementary Table 7). Of the 37 miRNAs that were DE in the pooled isoform analysis, 22 (59%) were also shown to be DE in this analysis. Two of these candidates included miR-16 and miR-32.

## Discussion

The measurement of circulating miRNAs has been explored in chronic disease states such as cancer, cardiovascular disease and some chronic viral infections such as HIV^25, 26^ and hepatitis C virus^27^. More recently, host miRNA profiles have been studied in response to acute viral infections associated with prolonged preclinical phases and/or high consequence of infection, such as rabies virus^28^ and Ebola virus^29^. The early identification of horses infected with HeV would provide an opportunity to decrease human exposure to infectious secretions and therefore reduce risk of zoonotic transmission. We have previously shown that HeV infection induces human host miRNA responses *in vitro*, and at least one of these responses, the transient up-regulation of miR-146a, is observed *in vivo* in horse blood. While miR-146a induction is not unique to HeV infection, it begged the question of whether altered expression patterns of blood miRNAs during the earliest stages of acute viral infection might reveal useful diagnostic biomarkers. As a first step in addressing this hypothesis, we have profiled horse miRNAs at the transcriptome-wide level in HeV-infected and uninfected horses. Following data filtering and normalization a panel of differentially expressed miRNAs that distinguished infected from uninfected animals was identified.

The collection of reference horse miRNAs in miRBase 21 were originally identified *in silico* ^30^, whilst horse miRNAs have since been detected *in vivo* ^31^. Our results confirm and extend the known horse miRNA transcriptome with the addition of a substantial number of putative novel miRNAs. The criteria and methods for identifying miRNAs vary widely, so it is anticipated that additional horse miRNAs will continue to be identified. The four highest-expressed miRNAs (miR-486-5p, miR-451, miR-16 and miR-92a) are known to be highly expressed in erythrocytes^32, 33^. Much effort has gone into identifying valid normalization strategies for miRNA quantification but consensus has not yet been achieved on the most suitable approach^34^. Many studies have used cell-free serum or plasma, or have used purified extracellular vesicles (exosomes). Other studies use whole blood, and there is not yet a consensus on which sample type is best. We observed in the horse blood that the two most abundant miRNAs (miR-486-5p, miR-451) were expressed at such high levels relative to the remaining miRNAs that they affected quantification of the raw data.

The development of miRNA profiling as a diagnostic tool requires further work to assess specificity. We cannot comment on whether changes to the host miRNA profile induced by HeV are similar or different to those induced by other pathogens. However, a comparison of our work to published studies reveal clear differences between the DE miRNAs induced by rabies virus, Ebola virus and HeV *in vivo*. On the other hand, miR-146a is up-regulated during acute stages of Ebola virus infection in humans and non-human primates and is consistent with several other viral infections, including HeV. Intriguingly, miR-146a was elevated in the blood of horses experimentally infected with HeV^16^ but showed no indication of upregulation in samples from infected horses analyzed in the present study. This potentially reflects the use of field samples taken during outbreaks, for which the time period between HeV exposure and blood collection is unknown. Another possibility is the dose difference, which may lead to different kinetics of miRNA regulation. In the original study, miR-146a was elevated in both horse and ferret blood within the first few days post-infection, but the effect is potentially transient, thus the collection of field samples may have missed the window for observing elevated miR-146a.

Host-encoded miRNAs responsive to viral infections can have large impacts on virus infection. We have recently shown that two immune-responsive miRNAs transiently up-regulated by HeV, miR-181 and miR-146a, promote HeV infection through different mechanisms. miR-181 strongly promotes HeV cellular entry by degrading ephrin receptors that bind and prevent viral attachment to the ephrin-B2 and –B3 HeV entry receptors^35^. miR-146a promotes HeV infection by degrading components of the A20 ubiquitination complex, which negatively regulates activity of NF-κB, a transcription factor that promotes HeV infection^16^. Multiple members of the let-7 family and miR-17~93 cluster are significantly elevated in HeV-infected horses (Fig. 5, Supplementary Tables 3 and 4). Cross-referencing results from a functional genomics screen highlights that let-7 family members inhibit HeV infection *in vitro*, while miR-17~93 members sharing the same seed sequence (AAGUG) promote HeV infection^35^. miR-16, validated in the present study as up-regulated by HeV, also shows a pro-viral phenotype^35^. Future work is required to define the mechanism of these miRNAs impacting the HeV infection cycle.

In conclusion our study demonstrates that HeV infection alters the expression profile of many host miRNAs. Whilst further work is required to optimize this technology, our study, and several others, suggest that host miRNA profiling during acute viral infections represents a novel approach to future diagnosis of infectious diseases.

## Methods

### Sample collection and processing

EDTA blood samples from horses were collected from horses that were naturally infected with HeV in New South Wales in 2011 and 2013, and in Queensland in 2011 (Table 1). Total RNA was harvested using Tri-reagent (Sigma, St. Louis, MO) following manufacturer’s instructions. Small RNA was separated from total RNA using the miRNEasy mini kit (Qiagen). Samples were prepared for Illumina next generation sequencing as previously described^20^. Sample preparation followed the “TruSeq Small RNA Library prep Kit” protocol (#15004197, Kit #RS-200-0012 from Illumina). Briefly, 10–50 ng of purified small RNA was used per sample. The 3′ adapter was ligated, followed by ligation of the 5′ adapter. RT and PCR were performed according to the protocol using 12 cycles of PCR. The small RNA yield was verified by running the samples on the Bioanalyzer HS chip (Agilent). Samples were pooled according to the concentration listed on the BA chip of the ~142 and 150 nt bands, to get an accurate yield of the adapter-ligated small RNA for each sample. The samples were gel purified using Novex 6% TBE gels (Invitrogen), run for 1 h at 145 V. After ethidium bromide staining for 2.5 minutes, bands between 145 and 160 bp were excised to account for the ~125 bp adapter. The gel was centrifuged through gel breaker tubes. 200 µL of ultrapure water was added to each tube and placed on a shaker at room temperature overnight. The gel debris was then run through a Qiagen spin column to elute the RNA. The libraries were finally concentrated by speed vac for 40 min, before verifying that the concentration was above the 2 nM yield needed for the run. The concentration and size was verified by Qubit HS (Invitrogen) and a BA HS chip. The libraries were denatured and clustered on the cBot according to cBot user guide (#15006165 Illumina). The sequencing was performed on the HiSeq. 2000 using v3 SBS reagents. Studies were approved by the CSIRO Australian Animal Health Laboratory’s Animal Ethics Committee and conducted following the Australian National Health and Medical Research Council Code of Practice for the Care and Use of Animals for Scientific Purposes guidelines for housing and care of laboratory animals.

### miRNA identification

Raw data were assessed for overall quality using FASTQC. Reads were trimmed of adaptors using Cutadapt, and subsequently filtered by length (reads <18 nt or >26 nt discarded) and quality (reads with >10% of bases with quality score <16 discarded) using the FASTX toolkit. MiRNA identification and quantification were carried out using miRDeep2^21^. Initially, trimmed and filtered reads from the six samples were combined and processed with the mapper.pl and mirdeep2.pl modules to identify the total pool of miRNAs present. Reference miRNA sequences were downloaded from the latest version of miRBase (version 21)^36^. For mirdeep2.pl, the following options were used: For the “mature_this_species” and “precursor_this_species” options, miRBase 21 horse mature and precursor sequences were used, while for the “mature_other_species” option, a file containing all miRBase 21 mature sequences from all mammals other than horse was supplied. Additional parameters provided to mirdeep2.pl were: –a 1, the minimum number of stacked reads for a precursor to be considered, and –b 1, the minimum miRDeep2 score for a novel miRNA to be retained. Consensus mature and star sequences and their corresponding precursors were extracted to FASTA format and compared against miRBase 21 mature reference sequences, allowing for minor differences in length.

### miRNA quantification

Quantitative miRNA expression in the individual samples was determined using the quantifier.pl module of the miRDeep2 package. Firstly, novel miRNAs identified in the pooled data (described above) were combined with miRBase 21 known horse miRNAs to construct the reference FASTA files. Quantifier.pl was then used to individually count the number of reads mapping to each miRNA in each individual sample. By default, individual reads were allowed to map at up to five different locations. Because of this behavior, the –W option was used to allocate counts fractionally across multiple mapping sites so that the sum of reported counts remained equal to the number of mapped reads. This was necessary to avoid distortion of the final count profiles due to a few highly-expressed sequences mapping to multiple loci. Individual count tables for each of the six samples were combined into a single count table in csv format and any miRNA that reported zero counts for every sample was discarded. Variance analysis was done by taking the coefficient of variation (CoV) (standard deviation of the six samples divided by the mean of the six samples for each individual miRNA). Graph smoothing was performed on the CoV plot using the exponentially-weighted moving averages method with a sliding window size of 15.

### miRNA normalization and differential expression (DE) testing

Normalization and DE testing were carried out using the R libraries EdgeR^23^ and DESeq2^22^. It should be noted here that since counts for multi-mapping reads were distributed fractionally across the mapping sites during the quantification step, many read counts reported non-integer values. Both EdgeR and DESeq2 require discrete (integer) counts, so all values were first rounded to the nearest integer. The following filters were then applied: (1) CPM (counts per million mapped reads) ≥1 in ≥3 samples, (2) Mean fold change ≥2, (3) FDR <0.05. In EdgeR, TMM normalization was applied, while DESeq2 used a default normalization method. Lists of significant DE miRNAs and normalized count tables were exported in csv format.

The second method used for DE analysis was based on miRNA ratios. MiRNAs with ≥100 reads in each replicate group (infected and uninfected) were tested against all other such miRNAs and ratios of raw counts for $\frac{miRNA\_A}{miRNA\_B}$ were determined for each of the six samples. Ratios with an average fold change ≥2 between infected and uninfected groups were subjected to unpaired t-tests. Multiple testing correction was applied using the Benjamini-Hochberg false discovery rate (FDR). Corrected p-values were sorted from lowest to highest, and exported to csv format.

### IsomiR analysis

For each unique read present in the trimmed and filtered data, per-sample counts were extracted using a custom python script that combined data from the *.reads and *.mrd files output by mapper.pl and mirdeep2.pl, respectively. For each mature and star miRNA locus reported by miRDeep2, the sequence with the highest read count was designated as the “most abundant isoform”. Conflicts (two or more sequences from the same locus with equally high read counts) were resolved according to the following rules: (1) Known sequences (100% identity to a mirbase miRNA) were given priority over novel sequences. (2) Sequences with zero mismatches relative to the horse genome were given priority over sequences containing mismatches. If conflicts were still present, the first remaining item was chosen. In a few instances where multiple loci had the same mature sequence they were collapsed into a single entry. This is because a mature miRNA can be encoded at more than one locus, and from the RNA-Seq data it is not possible to determine which of these loci the transcripts actually originated from, as they all report exactly the same read counts. For the isomiR analysis we ignored multiple copies and instead treated them as duplicates (i.e. keep one, discard the rest) by applying the rules described above. If conflicts remained, items were sorted alphabetically (by locus id) and the first remaining item was chosen. IsomiRs with ≤10 reads were discarded, then DE analysis was repeated as described above, but using most-abundant isoform counts instead of pooled isoform counts.

### Uridylation analysis

The most-abundant isoforms were compared with the consensus sequences and also the reference genome. Reads that did not conform to the reference were classified according to whether the differences were at either the 5′ or 3′ terminus or internal, and according to the observed mismatching nucleotide. A maximum of 1 mismatch per miRNA was present due to settings supplied to bowtie via miRDeep2 during read mapping. Python was used to analyze the total collection of individual reads to determine the proportion of mismatches of each type.

### Statistics

Differential expression analysis was carried out using DESeq2, which employs a generalized linear model based on the Negative Binomial (a.k.a. Gamma-Poisson) distribution, and Wald significance tests. False discovery rate was used for p-value correction. For PCR results, the difference between groups was analysed using a two-tailed Student’s *t* test, with a *P* value of <0.05 considered to be statistically significant. Error bars represent standard deviations, and all data points are the average of a minimum of 3 replicates.

## Electronic supplementary material

Supplementary Table 1

Supplementary Table 2

Supplementary Table 3

Supplementary Table 4

Supplementary Table 5

Supplementary Table 6

Supplementary Table 7
